# Supplementary material for: Spontaneous Bacterial Peritonitis: The Incremental Value of a Fast and Direct Bacterial Identification from Ascitic Fluids Inoculated in Blood Culture Bottles by MALDI-TOF MS for a Better Management of Patients
Source: Microorganisms. 2022 Jun 9;10(6):1188. doi: 10.3390/microorganisms10061188 (PMC9228703; doi:10.3390/microorganisms10061188)
Supplement: Supplementary file 1 [file microorganisms-10-01188-s001.zip › microorganisms-1747432-supplementary.pdf]

Table S1. Correct identifications from ascitic fluids on Day 0 (log(score) ≥ 1.5) of the bacterial species definitely identified on Day 1.

Definitive identification	Total no.	No. correct	% correct
Aerococcus urinae	**2**	2	100
Anaerococcus murdochii	**1**	0	0
Bacillus cereus	**1**	1	100
Bacteroides fragilis	**3**	3	100
Clostridium tertium	**1**	1	100
Corynebacterium amycolatum	**1**	0	0
Corynebacterium pseudodiphteriticum	**1**	1	100
Enterobacter aerogenes	**2**	1	50
Enterobacter cloacae	**11**	11	100
Enterococcus durans	**6**	4	67
Enterococcus faecalis	**9**	8	89
Enterococcus faecium	**9**	8	89
Enterococcus gallinarum	**1**	1	100
Escherichia coli	**24**	23	96
Klebsiella pneumoniae	**4**	3	75
Listeria monocytogenes	**5**	5	100
Moraxella osloensis	**2**	2	100
Providencia rettgeri	**3**	2	66
Propionibacterium acnes	**1**	0	0
Pseudomonas aeruginosa	**9**	5	56
Rothia mucilaginosa	**1**	0	0
Staphylococcus aureus	**9**	9	100
Staphylococcus capitis	**1**	1	100
Staphylococcus epidermidis	**23**	16	70
Staphylococcus haemolyticus	**4**	2	50
Staphylococcus hominis	**4**	3	75
Staphylococcus lugdunensis	**1**	1	100
Staphylococcus pasteuri	**2**	1	50
Staphylococcus warneri	**2**	2	100
Streptococcus anginosus	**2**	2	100
Streptococcus gallolyticus	**2**	1	50
*Streptococcus mitis group**	**5**	0	0
Streptococcus parasanguinis	**1**	1	100
Streptococcus pneumoniae	**3**	1	33

* *S. oralis* or *S. mitis* is defined as *S. mitis* group

Table S2. Clinical and microbiologic features of patients with polymicrobial SBP.

Patient	Gender	Age	Clinical features	First Antibiotic	Leukocytes count (cells/mm3)	GRAM on positive BCBs (D0)	Identification on BCBs (D0)	Antibiotic optimization (D0)	Identification on agar plate (D1)	Outcome
**Patient 2**	Female	75	SBP in a patient with HCV-related liver cirrhosis	Amoxicillin and clavulanic acid	400	Gram Negative rod	*E. cloacae*	Cefepime and metronidazole	*E. cloacae and P. aeruginosa*	Not favorable after 5 days of cefepime and metronidazole switch for imipenem.
**Patient 9**	Male	71	SBP and sepsis in a patient with ethylic liver cirrhosis.	Imipenem	1200	Gram positive cocci in chains and Gram-negative bacilli	*E. coli and E. faecium*	Addition of Daptomycin	*E. coli and E. faecium*	Not favorable. Patient died 7 days after appropriate antibiotic treatment
**Patient 16**	Male	37	SBP in a patient with ethylic liver cirrhosis and ulcerative colitis	Tazocillin	250	Gram positive cocci in chains and Gram-negative bacilli	*K. pneumoniae and E. faecalis*	No	*K. pneumoniae and E. faecalis*	Favorable. Patient received 6 days of Tazocillin switch for amoxicillin with ciprofloxacin
**Patient 17**	Male	60	SBP in a patient hospitalized for cholangiocarcinoma	Tazocillin	1500	Gram positive cocci in chains and Gram-negative bacilli	*E. cloacae*	No	*E. cloacae and E. durans*	Not favorable, Patient died 2 days after appropriate antibiotic treatment
**Patient 18**	Male	63	SBP in a patient hospitalized for ruptured esophageal varices	Cefotaxime	22140	Gram positive cocci in chains and Gram-positive in cluster	*S. pneumoniae*	No	*S. pneumoniae and Rothia mucilaginosa*	Patient received 4 days of Cefotaxime. After two recurrences of SBP, the patient had a favorable outcome
